# A Nanopore Phosphorylation Sensor for Single Oligonucleotides and Peptides

**DOI:** 10.34133/2019/1050735

**Published:** 2019-11-04

**Authors:** Yi-Lun Ying, Jie Yang, Fu-Na Meng, Shuang Li, Meng-Ying Li, Yi-Tao Long

**Affiliations:** ^1^State Key Laboratory of Analytical Chemistry for Life Science, School of Chemistry and Chemical Engineering, Nanjing University, Nanjing 210023, China; ^2^Chemistry and Biomedicine Innovation Center, Nanjing 210023, China; ^3^School of Chemistry and Molecular Engineering, East China University of Science and Technology, Shanghai 200237, China

## Abstract

The phosphorylation of oligonucleotides and peptides plays a critical role in regulating virtually all cellular processes. To fully understand these complex and fundamental regulatory pathways, the cellular phosphorylate changes of both oligonucleotides and peptides should be simultaneously identified and characterized. Here, we demonstrated a single-molecule, high-throughput, label-free, general, and one-step aerolysin nanopore method to comprehensively evaluate the phosphorylation of both oligonucleotide and peptide substrates. By virtue of electrochemically confined effects in aerolysin, our results show that the phosphorylation accelerates the traversing speed of a negatively charged substrate for about hundreds of time while significantly enhances the translocation frequency of a positively charged substrate. Thereby, the kinase/phosphatase activity could be directly measured with the aerolysin nanopore from the characteristically dose-dependent event frequency of the substrates. By using this straightforward approach, a model T_4_ oligonucleotide kinase (PNK) further achieved the nanopore evaluation of its phosphatase activity and real-time monitoring of its phosphatase-catalyzed dephosphorylation at a single-molecule level. Our study provides a step forward to nanopore enzymology for analyzing the phosphorylation of both oligonucleotides and peptides with significant feasibility in fundamental biochemical researches, clinical diagnosis, and kinase/phosphatase-targeted drug discovery.

## 1. Introduction

Phosphorylation plays a ubiquitous and essential role in regulating almost all biological functions. The level of oligonucleotide and peptide phosphorylation is well modulated by both kinases and phosphatases [1–5]. Although the importance of oligonucleotide/peptide phosphorylation has long been recognized, an appreciation for the complex and fundamental role of phosphorylation state is still required [6, 7]. The multiple phosphorylation of oligonucleotide/peptide works together to contribute to the outcome of the signaling pathway. To fully understand this complex and fundamental regulatory process, the phosphorylation changes of both oligonucleotides and peptides must be identified and characterized rapidly. Although a variety of methods have been developed to assess the evaluation of oligonucleotide/peptide phosphorylation, including radiometric assays [8], fluorescence [9, 10], chemiluminescence [11], electrochemistry [12, 13], surface plasmon resonance [14], mass spectrometry [15], and nano-CT imaging [16], these methods are only effective to either oligonucleotide or peptide phosphorylation. Moreover, the present methods suffer from harmful radioactive labels, sophisticated and costly fluorescence labels, multistep detection procedures, inadequate contact in reactants, and expensive recognition proteins. These drawbacks limit the generalization of the method to be appropriate for evaluations of the phosphorylation state in the cases of both oligonucleotides and peptides. Much less, the monitoring of the phosphorylation also requires a single-molecule approach for the identification of a rare population of phosphorylated substrates from the native substrates. Therefore, it is still challenging but highly desirable to develop a simple, convenient, label-free, and generalized sensor for phosphorylation detection on both oligonucleotides and peptides.

**Figure 1:**
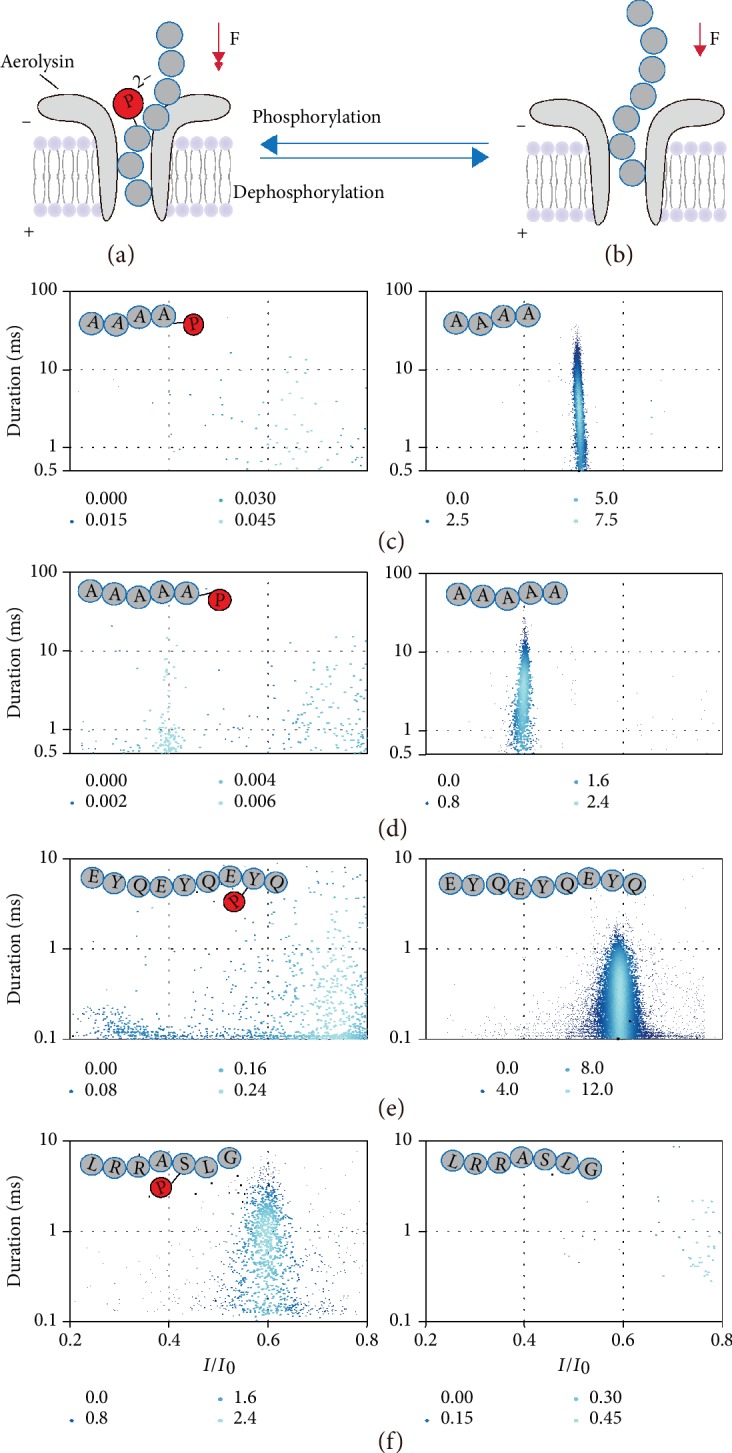
An aerolysin nanopore sensor for the phosphorylation detection of both oligonucleotides and peptides. (a) The aerolysin nanopore sensing of the phosphorylated oligonucleotide/protein. The phosphorylated substrate with additional negative charges suffers the strong electrophoretic force (red arrow) through the aerolysin nanopore. (b) The aerolysin nanopore sensing of the native oligonucleotide/peptide. The native substrate bears a weaker electrophoresis force (red arrow) to transverse through the aerolysin nanopore compared to the phosphorylated substrate. The scatter plots for the aerolysin nanopore sensing of poly(dA)_4_ (c), poly(dA)_5_ (d), EYQEYQEYQ peptide (e), and LRRASLG peptide (f). The phosphorylated and native substrates are shown in the left column and right column, respectively. Each point in the scatter plots is colored by the Kernel Density. The large points with light color represent the high spatial density of nearby points. The data for substrates in (c), (d), and (e) were acquired at the applied voltage of +120 mV, in 1 M KCl, 10 mM Tris, and 1.0 mM EDTA buffer at pH 8.0 in the presence of 2.0 *μ*M substrates. The data for substrates in (f) were acquired at the applied voltage of +120 mV, in 1 M KCl, 50 mM Tris, and 20 mM MgCl_2_ buffer at pH 7.5 in the presence of 5.0 *μ*M substrates. All the ionic currents were filtered at 5 kHz and sampled at 100 kHz. The mass spectra of all the phosphorylated and native substrates are shown in [Supplementary-material supplementary-material-1].

A single biological membrane protein-based single-biomolecule interface provides a single-molecule nanopore platform for sensitive detection of DNA/RNA [17–22], peptides [23–25], proteins [26–29], enzymes [30, 31], and host-guest molecules [32, 33]. The confined nanopore interface effectively captures a single molecule from the bulk solution at an applied potential, resulting in the typical ionic blockages for each analyte. Although previous nanopore studies evaluate the phosphorylation of the peptides, it requires genetic engineering of the peptides into the biological nanopores and/or chemical modification of the leading DNA sequence on the model peptides [34–37]. Moreover, the previous nanopore sensing interface (e.g., *α*-hemolysin) exhibits less sensitivity for directly identifying the phosphorylation states of oligonucleotides through the reading of ionic current. Therefore, an efficient nanopore sensing interface with robust interactions with the phosphate groups of oligonucleotides/peptides is urgently demanded for directly distinguishing the phosphorylation from the native substrates. An aerolysin nanopore owns an extremely strong sensing interface to interact with charged oligonucleotides [38–40] and peptides [24, 25], which realizes the discrimination of a single-nucleotide difference of oligonucleotides as well as the length and charge difference of the peptides. Among all the reported biological nanopores, the aerolysin shows the largest effective charge value for confining the oligonucleotides/peptides inside its *β*-barrel without the needs of motor proteins or labels [25]. Herein, we employed the electrochemical confinement of the aerolysin nanopore to achieve general and feasible nanopore phosphorylation sensors of both oligonucleotides and peptides (Figure 1(a) and (b)). Our statistical results of oligonucleotides and peptides demonstrate that the phosphorylation significantly regulates their traversing speed for about hundreds of time, which could be directly read from the duration as well as the frequency of ionic current signatures (Figure 1(c)–(f)). We further selected the substrate oligonucleotide sequence of poly(dA)_4_ for the model T4 oligonucleotide kinase (PNK) to achieve the proof-of-concept experiments which are aimed at label-free evaluation of phosphorylation/dephosphorylation-related enzyme activity with nanopore methods. PNK plays a critical role in a majority of normal cellular events, including DNA recombination, DNA replication, and the repair of DNA during strand interruption [41, 42]. Our results demonstrate that one-step strategy is successfully applied to monitor the phosphatase-catalyzed dephosphorylation of the oligonucleotide at a single-molecule level. Our approach presents significant advantages over traditional kinase/phosphatase assays [9, 43]. Therefore, the presented nanopore sensor shows the significant efficient for characterizing the phosphorylation of both oligonucleotides and peptides, which is potentially beneficial for the fundamental biochemical researches, clinical diagnosis, and kinase/phosphatase-targeted drug discovery.

## 2. Results

### 2.1. Phosphorylate Evaluation of Both Oligonucleotide and Peptide Substrates

As shown in Figure 1(c), we employed the aerolysin sensing interface to identify both the oligonucleotides and peptides' phosphorylate state. As for the proof of concept for studying the aerolysin sensing ability of oligonucleotide phosphorylation, poly(dA)_4_ (5′-AAAA-3′) and poly(dA)_5_ (5′-AAAAA-3′) were chosen as the model oligonucleotide substrates. First, the poly(dA)_4_ and poly(dA)_5_ were added into the cap side of the aerolysin nanopore. The applied potential was set at +120 mV while the cap side was defined as a virtual ground. Both poly(dA)_4_ and poly(dA)_5_ without phosphorylation illustrate one prominent translocation population in the scatter plots of the duration and residual current ratio (*I*/*I*_0_) (Figure 1(c) and (d), right). Here, *I* represents the residual current, while *I*_0_ stands for the open pore current. *I*/*I*_0_ reflects the residual current depth. These results are consistent with our previous finding that the translocation of negatively charged poly(dA)_*n*_ (3 ≤ *n* ≤ 10) produces the sharp distribution of blockage current with duration time as long as tens of milliseconds [38]. Since the poly(dA)_5_ owns one nucleotide longer than poly(dA)_4_, it leads to a larger current blockage amplitude with a statistical *I*/*I*_0_ of 0.39. After the phosphorylation at 3′ end, the poly(dA)_4_-3′-P hardly produces the blockages longer than 0.5 ms (Figure 1(c), left). Similarly, the phosphorlation of poly(dA)_5_-3′-P significantly decreases the statistical translocation duration from 3.6 ms to less than 1 ms (Figure 1(d), left). Since the addition of the phosphate group at the 3′ terminal of the oligonucleotide provides the additional two negatively charges, it induces the stronger electrophoresis force acting on the oligonucleotides. Therefore, the phosphorylated oligonucleotides behave at a faster translocation speed through the aerolysin nanopore than the native oligonucleotide. The length-dependent translocation speed is accelerated for both poly(dA)_4_-3′-P and poly(dA)_5_′-3′-P. The detail mechaniusms of the phosphorylation-driven acceleration are currently under studying in our group.

Unlike the oligonucleotide carriers of uniform negative charges, the peptides always bear nonuniform negative and positive charges. Therefore, the nanopore phosphorylation sensors for peptides not only should be sensitive to the phosphorylation of negatively charged peptide but also are supposed to generate the distinguishable blockage differences for the phosphorylation of positively charged peptide. To demonstrate the sensing ability of the aerolysin nanopore, we first used a peptide sequence of (EYQ)_3_ with three negatively charged glutamic acids. Figure 1(e) shows that this peptide generates a distinctive population with Gaussian current peak value of *I*/*I*_0_ = 0.53 and statistical fitted duration of 0.25 ms. This result is consistent with our previous study [25] that the negatively charged peptide experiences a translocation process through the aerolysin nanopore under positive potential. Similar to the identification of phosphorylated oligonucleotide, the phosphorylation of Tyr-8 provides two additional negative charges to the peptide of (EYQ)_3_, which enhances the electrophoresis force for a fast peptide translocation. Therefore, the rapid translocation of phosphorylated (EYQ)_3_ hardly shows any blockages due to the bandwidth limitation of the amplifier. (Figure 1(e), left). This distinguishable differences on the event durations and frequencies between (EYQ)_3_ and phosphorylated (EYQ)_3_ demonstrate that the aerolysin nanopore could be extended to the evaluation of the phosphorylation for the negatively charged peptide. Then, we further study the aerolysin sensing ability of positively charged peptide with and without phosphorylation. Under the applied potential of +120 mV, the positively charged peptide of LRRASLG hardly enters into the aerolysin nanopore, resulting in few blockages (Figure 1(f)). After phosphorylation of Ser-5, the net charge of the peptide decreases from +2 to 0 at pH = 7.5, leading to the enhanced driving force at +120 mV. Consequently, the number of blockages obviously increases after the phosphorylation of LRRASLG, which shows a Gaussian peak value of *I*/*I*_0_ = 0.59 and exponential fitted duration of 1.2 ms. Overall, the phosphorylation of the oligonucleotides and peptides increases the carrying negative charges of the substrate, which leads to the distinctive regulation of the driving force for the translocation through the aerolysin nanopore. As to the negatively charged oligonucleotides and peptides, the phosphorylation accelerates the translocation speed of the substrate, resulting in the significant decrease of the duration time for the main distribution of the blockages. Since the majority of the phosphorylated oligonucleotides/peptides with short durations are below the bandwidth of amplifier (5 kHz), the blockage frequency in the main distribution would be decreased accordingly. As to the positively charged peptide, the phosphorylation allows it to enter and then interact with the *β*-barrel of the aerolysin. Therefore, the phosphorylation of positively charged peptide “turns on” the characteristic blockages in the main distribution. Note that the difference of the duration time and frequency depends mainly on the initial net charges and the length of the native oligonucleotide/peptide. Moreover, previous studies showed [24, 25, 44] that the limit of detection for nanopore sensing of short oligonucleotide poly(dA)_*n*_ (3 ≤ *n* ≤ 10) could reach to as low as 1.0 × 10^−13^ M while that for the short peptide is expected to a nanomolar level. The high capture rate of both oligonucleotides and peptide ensures the low limit of detection for the phosphorylation sensing without amplification methods. Therefore, the aerolysin nanopore could achieve direct and label-free discrimination of single-molecule phosphorylation of both oligonucleotides and peptides via monitoring the frequency and duration responses from the characteristic blockages.

### 2.2. Direct PNK Activity Evaluation Using the Feasible Aerolysin Nanopore

**Figure 2:**
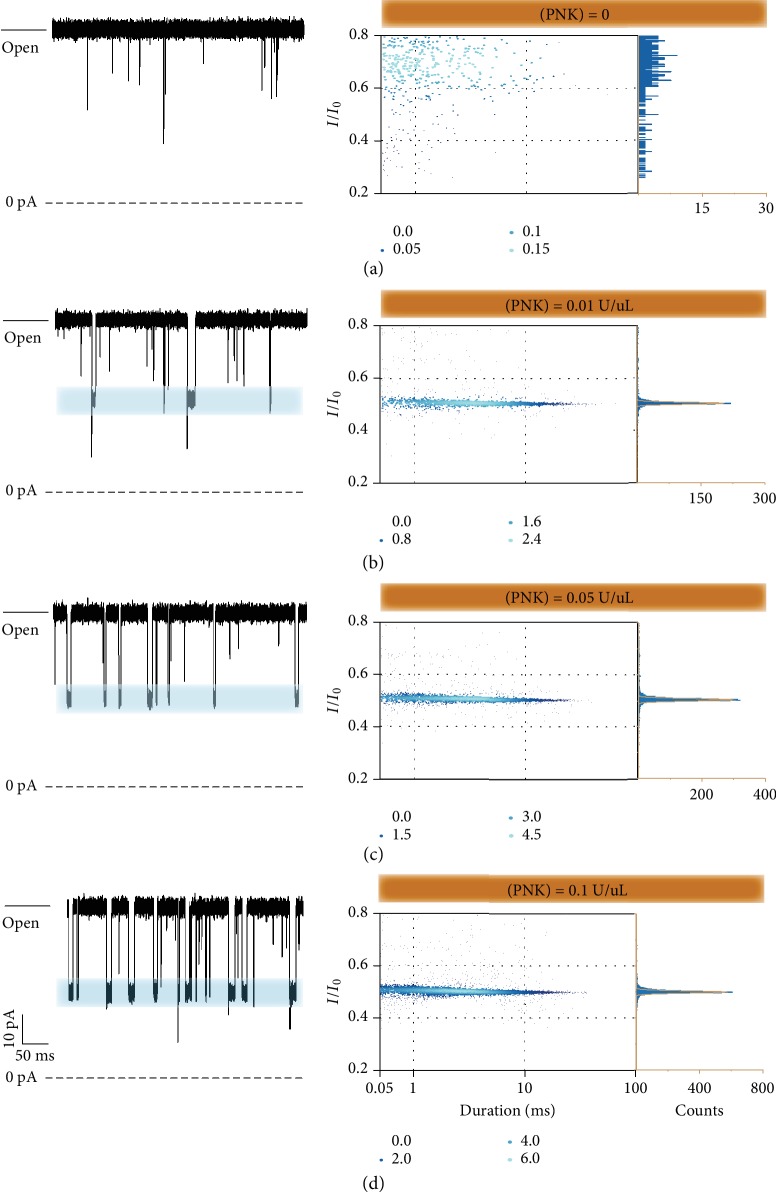
(a–d) The raw current trace (left), scatter plots (middle), and relevant *I*/*I*_0_ histogram (right) of the reaction solution detected by the aerolysin nanopore with the absence (a) and in the presence of different concentrations of PNK: 0.01 U/*μ*L (b), 0.05 U/*μ*L (c), and 0.1 U/*μ*L (d). Each point in the scatter plots is colored by the Kernel Density. The width of the band in the raw current trace was calculated according to the current Gaussian peak width of the reaction solution ([Supplementary-material supplementary-material-1]). All the experiments were performed at +120 mV and filtered at 5 kHz with a sampling rate of 100 kHz.

The kinase and phosphatase could induce the phosphorylation and dephosphorylation of the oligonucleotide/peptide. Based on the above mechanism, one could expect to monitor the frequency responses of characteristic blockages from the oligonucleotide/peptide substrate to evaluate single-molecule kinase/phosphatase activity with this general aerolysin nanopore sensor. For the proof of concept, we further select PNK as a model system. In the presence of Mg^2+^, PNK can remove the 3′phospotase group of poly(dA)_4_-3′-P [41, 45]. Then, the assays for assessing PNK activity were performed as follows: Briefly, 100 *μ*L reaction solution containing 100 *μ*M poly(dA)_4_-3′-P and 10 mM MgCl_2_ was incubated with a certain concentration of PNK at 37°C. The reaction was quenched by heating to 65°C for 20 min to denature the enzyme after incubation for 1 h. Full experimental details can be found in Supplementary Information. The reaction solution was premixed with 900 *μ*L electrolyte solution and then added into the cap side chamber. After the insertion of the aerolysin nanopore to the lipid bilayer, the analyzed solution containing 100 *μ*M poly(dA)_4_-3′-P without PNK hardly induces the typical current blockages with a long duration at +120 mV (Figure 2(a)) while the typical blockage events emerged evidently in the presence of PNK as expected (Figure 2b–d). The PNK catalysis products of A_4_ generates typical blockage events, which were mainly distributed in the target distribution with duration time ranging from 1 to 100 ms and *I*/*I*_0_ falling between 0.4 and 0.6 (Figure 1(e)). The enlargement of the typical blockage events is shown in [Supplementary-material supplementary-material-1].

To probe the relationship between the PNK concentrations and its catalysis-induced event frequencies, the different concentrations of PNK ranging from 0.01 to 0.1 U/*μ*L were successively added into the cap side of the aerolysin nanopore. Here, we defined the frequency of typical blockage events in the target distribution as *f*_r_. When the concentration of PNK increases from 0.01 to 0.1 U/*μ*L, the *f*_*r*_ increases from 14.47 ± 0.04 s^−1^ to 46.18 ± 0.13 s^−1^. There exists a positive correlation between PNK concentration and *f*_*r*_ ([Supplementary-material supplementary-material-1] and [Supplementary-material supplementary-material-1]). The strategies which could be further used to enhance the capture rate include optimizing the concentration gradient and the types of electrolytes [46, 47], reducing the volume of the chamber, and designing the mutagenized aerolysin [48]. Therefore, it is possible to achieve a much lower detection limit compared to other various sensitive PNK detection methods.

### 2.3. Real-Time Monitoring the PNK-Catalyzed Dephosphorylation

**Figure 3:**
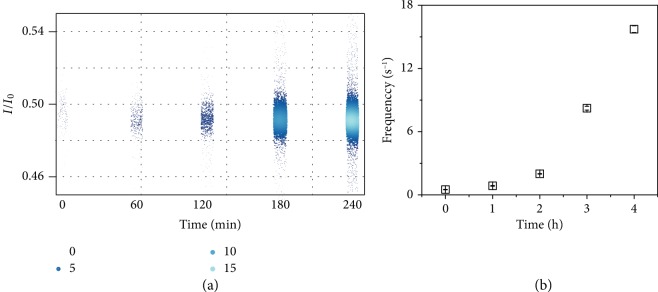
(a) *I*/*I*_0_ versus recording time (min) for the PNK-catalyzed dephosphorylation process of poly(dA)_4_. The color and the size of each point represent the Kernel Density of the typical events from the target distribution in which the *I*/*I*_0_ is located in 0.4~0.6 and the duration is larger than 1 ms. (b) Frequency of typical events versus time for the PNK dephosphorylation process. The values of the frequency were calculated using data of the first 8 minutes of each 60 minutes. The related scatter plots are shown in [Supplementary-material supplementary-material-1]. The data were recorded at +120 mV and filtered at 5 kHz with a sampling rate of 100 kHz. The reaction temperature was 30 ± 1°C.

To perform the real-time monitoring of the PNK-catalyzed dephosphorylation, the enzymatic catalysis was directly carried out in the cap side compartment containing 20 *μ*M poly(dA)_4_-3′-P and 10 mM MgCl_2_. Hardly, any typical blockage events were observed before the addition of PNK to the chamber ([Supplementary-material supplementary-material-1]). After the addition of 0.25 U/*μ*L PNK, the typical blockage events are clearly observed (Figure 3(a)). According to the statistical analysis, the typical blockage events are also distributed in the target region in which the *I*/*I*_0_ is between 0.4 and 0.6, and the duration is longer than 1 ms ([Supplementary-material supplementary-material-1]). Moreover, as shown in Figure 3(b), the frequency of the typical events increases with the recording time. Since the active PNK scarcely induces any interfere, the variation of the typical blockage event frequency here is resulted from the increase of the catalyzed product poly(dA)_4_ in the cap chamber. Thus, this result evidences the feasibility of aerolysin nanopore for achieving a real-time recording of the enzyme-catalyzed dephosphorylation process, which allows the potential monitoring of phosphorylation/dephosphorylation kinetics at the single-molecule level.

## 3. Discussions

These above mechanisms and observed results demonstrate that the aerolysin sensor reported here could achieve label-free and general evaluation of the phosphorylation of oligonucleotides and peptides, leading to an accurate quantification of the corresponding enzyme catalysis activity. The advantages of the aerolysin nanopore strategy show but not limit to the following charming advantages: (1) the strong ability for the sensitive response to the charge states of both DNA and peptide substrates thereby directly regulates the event duration as well as the frequency without exogenous labels, immobilization, and cooperative enzymes. Employing this generalized nanopore approach, one has the potential to characterize the complex regulatory process resulting from both DNA and protein kinases/phosphatases. (2) Few ionic responses to the background [49] ensure the potential of the aerolysin nanopore not only to screen large-scale inhibitors but also to direct assay kinase/phosphatase activity in cell lysate. This feature provides the possibility for the aerolysin nanopore in intracellularly analyzing the multiple kinase and phosphatase functions. (3) Long-term stability and high reproducibility ensure the long-time monitoring of the kinase/phosphatase activity. In further combination with solid-state material (e.g., quartz nanopipette) for supporting aerolysin nanopore, the aerolysin nanopore may eventually achieve the single cell evaluation of kinase/phosphatase.

We could anticipant that our generalized strategy facilitates the understanding of phosphorylation-involved cellular signaling pathway. Moreover, the diagnostic application would build upon our initial findings with the further improvements of clinical sampling system (e.g., parallel microfluidics chip).

## Supplementary Materials

Supplementary Materials Supplementary Information: materials, nanopore formation and data collection, and procedures for detection of PNK activity. Figure S1: mass spectrometry characterization of poly(dA)4 (a) and poly(dA)4-3′-P (b). The calculated values of *m*/*z* for poly(dA)4 and poly(dA)4-3′-P are 1190.90 and 1270.90, respectively. Figure S2: mass spectrometry characterization of poly(dA)5 (a) and poly(dA)5-3′-P (b). The calculated values of *m*/*z* for poly(dA)5 and poly(dA)5-3′-P are 1504.11 and 1584.11, respectively. Figure S3: mass spectrometry characterization of EYQEYQEYQ (a) and EYQEYQEpYQ (b). The calculated values of *m*/*z* for EYQEYQEYQ and EYQEYQEpYQ are 1279.29 and 1359.29, respectively. Figure S4: mass spectrometry characterization of LRRASLG (a) and LRRApSLG (b). The calculated values of *m*/*z* for LRRASLG and LRRApSLG are 771.92 and 851.92, respectively. Figure S5: the raw current trace induced by the PNK reaction system in the presence of 0.1 U/*μ*L PNK and 100 *μ*M poly(dA4)-3′-P. The enlargement of typical blockage event is shown in the right. The events were recorded in the buffer solution of 1.0 M KCl and 10 mM Tris (pH = 8) at +120 mV. Figure S6: the frequency histogram of PNK-catalyzed reaction solution for the PNK concentration of 0.01 U/*μ*L (a), 0.05 U/*μ*L (b), and 0.1 U/*μ*L (c) in the presence of 100 *μ*M poly(dA4)-3′-P. The data was recorded in the 0-5 min (up), 5-10 min (middle), and 10-15 min (bottom), respectively. The event frequency was calculated by *f*_*r*_ = 1/ton, where ton was the interval time between the consecutive typical blockage events. The histograms of frequency were fitted into the exponential equations. Figure S7: the histogram of *I*/*I*_0_ of poly(dA)4 (a) and the PNK-catalyzed events (b). The histograms of *I*/*I*_0_ were fitted into the Gaussian equations, and their peak widths at half height were *I*/*I*_0_ = 0.01. Figure S8: the raw data (a) and relative scatter plots (b) before adding the PNK in the real-time monitoring of PNK-catalyzed dephosphorylation assay. The recording time of data in scatter plots is 5 minutes. Figure S9: the scatter plots for real-time monitoring PNK dephosphorylation process by the aerolysin nanopore at the recording time of 0 h (a), 1 h (b), 2 h (b), 3 h (c), 4 h (d), and 5 h (e). Each scatter plots contain the statistical data of 8 minutes. Each point in the scatter plots is colored by the Kernel Density. 250 units of PNK and 100 *μ*M poly(dA4)-3′-P were added into the cap side chamber which was filled with detection buffer (1.0 M KCl, 10 mM MgCl_2_, and 10 mM Tris; pH = 6). The temperature was controlled at 30 ± 1°C, and the recorded potential was set at +120 mV. Table S1: the event frequencies (*f*_*r*_) induced by the reaction buffer containing different PNK concentrations.
