# Supplementary material for: A Nanopore Phosphorylation Sensor for Single Oligonucleotides and Peptides
Source: Research (Wash D C). 2019 Nov 4;2019:1050735. doi: 10.34133/2019/1050735 (PMC6944226; doi:10.34133/2019/1050735)
Supplement: Supplementary Materials — Supplementary Information: materials, nanopore formation and data collection, and procedures for detection of PNK activity. Figure S1: mass spectrometry characterization of poly(dA)4 (a) and poly(dA)4-3′-P (b). The calculated values of m/z for poly(dA)4 and poly(dA)4-3′-P are 1190.90 and 1270.90, respectively. Figure S2: mass spectrometry characterization of poly(dA)5 (a) and poly(dA)5-3′-P (b). The calculated values of m/z for poly(dA)5 and poly(dA)5-3′-P are 1504.11 and 1584.11, respectively. Figure S3: mass spectrometry characterization of EYQEYQEYQ (a) and EYQEYQEpYQ (b). The calculated values of m/z for EYQEYQEYQ and EYQEYQEpYQ are 1279.29 and 1359.29, respectively. Figure S4: mass spectrometry characterization of LRRASLG (a) and LRRApSLG (b). The calculated values of m/z for LRRASLG and LRRApSLG are 771.92 and 851.92, respectively. Figure S5: the raw current trace induced by the PNK reaction system in the presence of 0.1 U/μL PNK and 100 μM poly(dA4)-3′-P. The enlargement of typical blockage event is shown in the right. The events were recorded in the buffer solution of 1.0 M KCl and 10 mM Tris (pH = 8) at +120 mV. Figure S6: the frequency histogram of PNK-catalyzed reaction solution for the PNK concentration of 0.01 U/μL (a), 0.05 U/μL (b), and 0.1 U/μL (c) in the presence of 100 μM poly(dA4)-3′-P. The data was recorded in the 0-5 min (up), 5-10 min (middle), and 10-15 min (bottom), respectively. The event frequency was calculated by fr = 1/ton, where ton was the interval time between the consecutive typical blockage events. The histograms of frequency were fitted into the exponential equations. Figure S7: the histogram of I/I0 of poly(dA)4 (a) and the PNK-catalyzed events (b). The histograms of I/I0 were fitted into the Gaussian equations, and their peak widths at half height were I/I0 = 0.01. Figure S8: the raw data (a) and relative scatter plots (b) before adding the PNK in the real-time monitoring of PNK-catalyzed dephosphorylation assay. The recording ti [file 1050735.f1.docx]

**Supplementary Information**

A Nanopore Phosphorylation Sensor for Singles Oligonucleotide and Peptides

Yi-Lun Ying^#1^, Jie Yang^#2^, Fu-Na Meng^2^, Shuang Li^2^, Meng-Ying Li^1^, and Yi-Tao Long^*1^

^1^ State Key Laboratory of Analytical Chemistry for Life Science, School of Chemistry and Chemical Engineering, Nanjing University, Nanjing, 210023, P. R. China

^2^ School of Chemistry and Molecular Engineering, East China University of Science and Technology, Shanghai, 200237, P. R. China.

* To whom correspondence should be addressed: yitaolong@nju.edu.cn;

^#^ These two authors contribute equally to this work

Materials

All aqueous solutions for analytical studies were prepared with ultrapure water (reaching a resistivity of 18.2 MΩ·cm at 25 °C) from the Milli-Q System (EMD Millipore, Billerica, MA, USA). 1,2-Diphytanoly-sn-glycero-3-phosphocholine (powder) was obtained from Avanti Polar Lipids Inc, Alabaster, AL, USA. All the peptides and phosphorylated peptide were synthesized by GL Biochem Ltd. (Shanghai, China). All polynucleotide and phosphorylated polynucleotide samples used in experiments were synthesized and purified by Sangon BiotechCo., Ltd. (Shanghai, China). The T4 Polynucleotide Kinase (PNK) catalytic subunit was purchased from New England Biolabs (Ipswich, MA, USA). The detailed experimental methods of producing the wild-type aerolysin are described in our previous studies [1, 2]. All the other reagents were of analytical grade and were used as received without further purification. The mass spectra of all the polynucleotides and peptides are shown in Figure S1-S4.

Nanopore Formation and Data Collection

The detailed experimental methods of forming the wild-type aerolysin nanopore are described in our previous studies [1-4]. Briefly, the two chambers beside the nanopore were filled with the buffer solution (1 M KCl, 10 mM MgCl_2_ and 10 mM Tris at pH 8). The experiments were conducted with Axon 200B amplifier (Molecular Devices, CA, USA) and converted into digital data with Digidata 1440 A A/D converter (Molecular Devices, CA, USA). The internal low-pass Bessel filter of the amplifier was set to 5 kHz. The data were recorded by Clampex 10.4 software (Molecular Devices, CA, USA). Data analysis was performed using a homemade data analysis software PyNano (https://decacent.github.io/PyNano/) and MOSAIC software [5], and Origin 9.0 (OriginLab Corporation, Northampton, MA, USA). The kernel density evaluation was achieved by the package of scipy in python.

Procedures for Detection of PNK Activity

The PNK-catalyzed dephosphorylation reaction was carried out in a 100 μL volume of reaction solution (1 M KCl, 10 mM MgCl_2_, 10 mM Tris, pH = 6) containing 100 μM poly(dA_4_)-3’-P and a certain concentration of PNK at 37 ℃ for 1 h. Afterward, the reaction system was immediately put into water bath with the constant temperature of 65 ℃ for 20 min to denature the enzyme. Then, the reaction solution was premixed with 900 μL buffer solution (1 M KCl, 10 mM Tris) and then added into the cap side chamber of aerolysin nanopore, after it restored to the room temperature. The current traces of aerolysin nanopore were recorded for analyzing the reaction system.


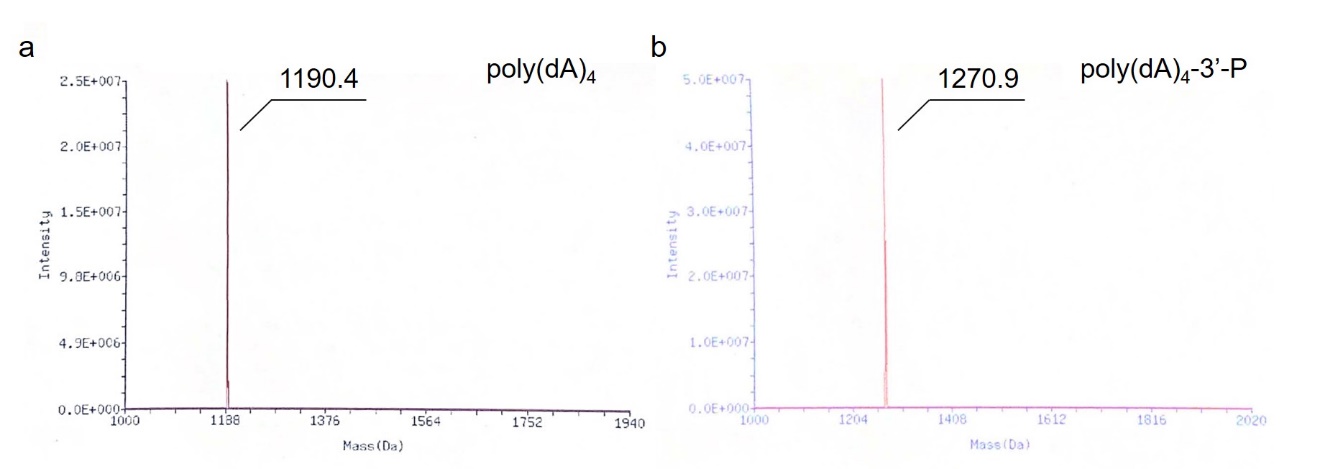


**Figure S1.** Mass spectrometry characterization of poly(dA)_4_ (a) and poly(dA)_4_-3’-P (b). The calculated values of m/z for poly(dA)_4_ and poly(dA)_4_-3’-P are 1190.90 and 1270.90, respectively.


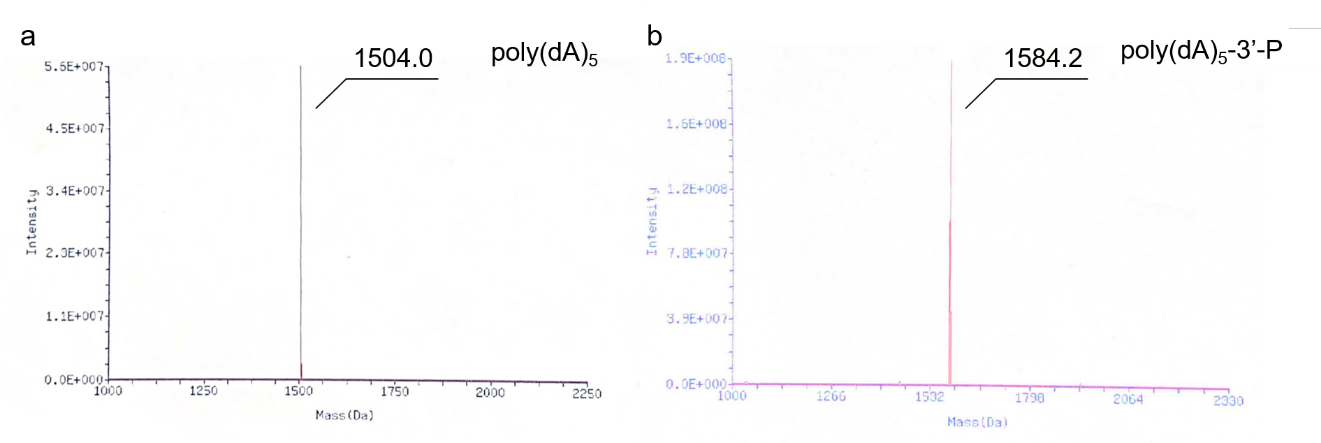


**Figure S2.** Mass spectrometry characterization of poly(dA)_5_ (a) and poly(dA)_5_-3’-P (b). The calculated values of m/z for poly(dA)_5_ and poly(dA)_5_-3’-P are 1504.11 and 1584.11, respectively.


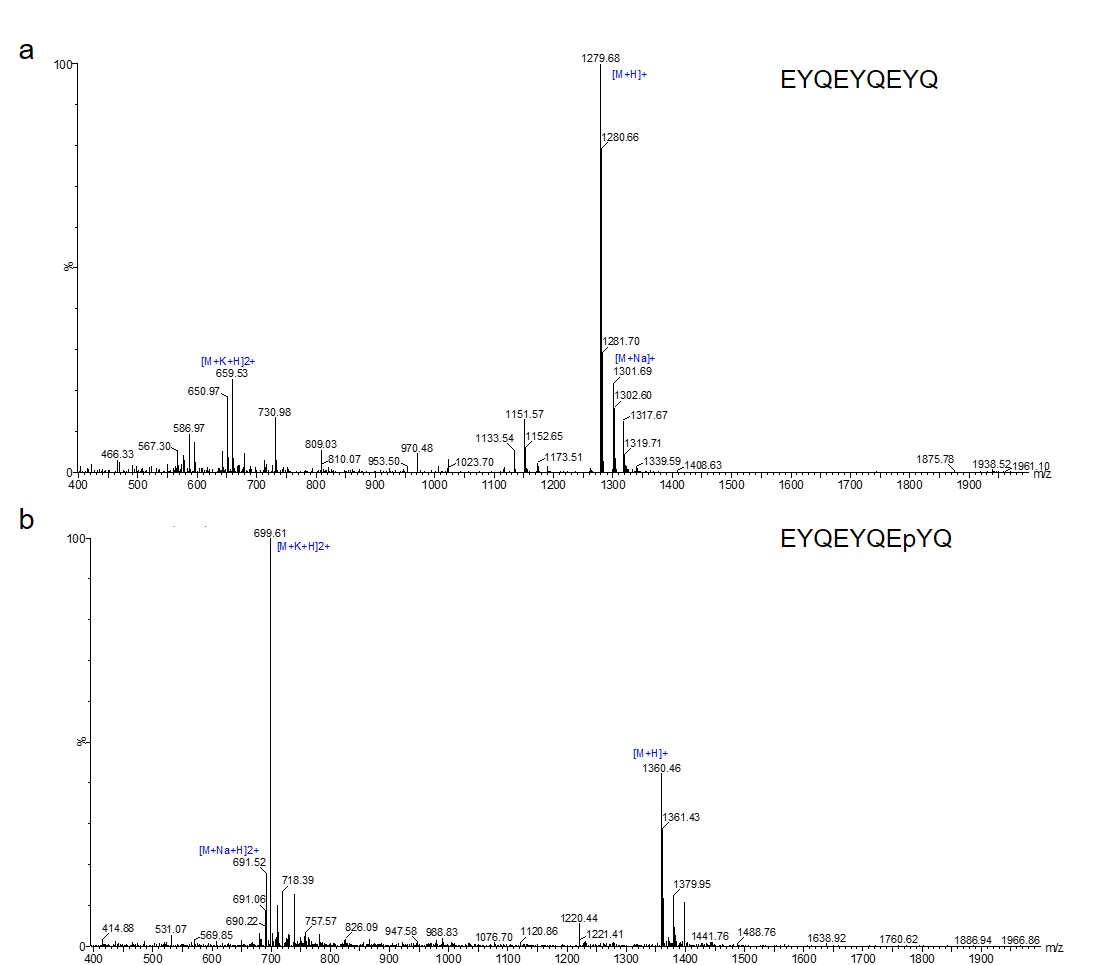


**Figure S3.** Mass spectrometry characterization of EYQEYQEYQ (a) and EYQEYQEpYQ (b). The calculated values of m/z for EYQEYQEYQ and EYQEYQEpYQ are 1279.29 and 1359.29, respectively.


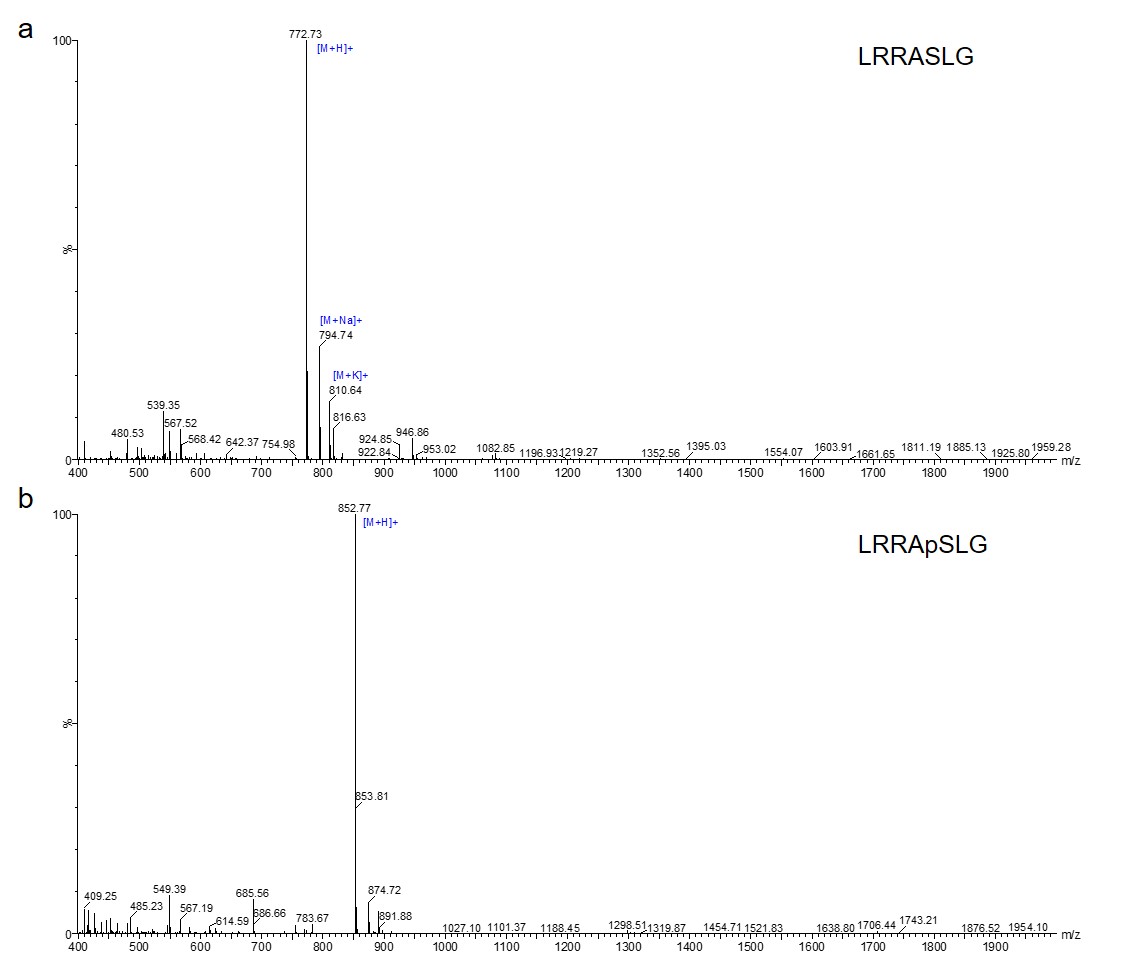


**Figure S4.** Mass spectrometry characterization of LRRASLG (a) and LRRApSLG (b). The calculated values of m/z for LRRASLG and LRRApSLG are 771.92 and 851.92, respectively.


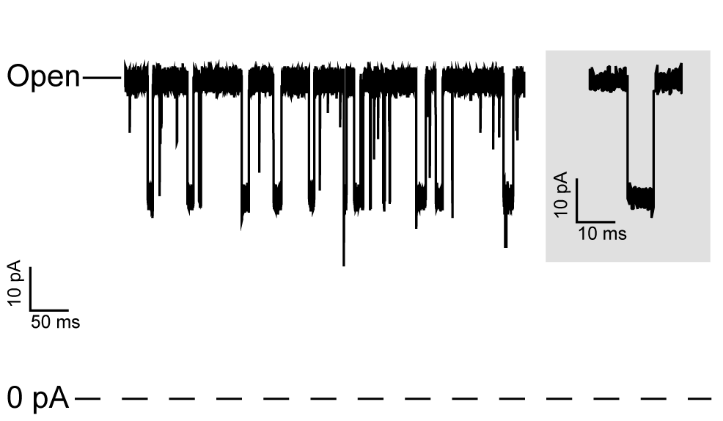


**Figure S5.** The raw current trace induced by the PNK reaction system in the presence of 0.1 U/μL PNK and 100 μM poly(dA_4_)-3’-P. The enlargement of typical blockage event is shown in the right. The events were recorded in the buffer solution of 1.0 M KCl, 10 mM Tris (pH=8) at +120 mV.


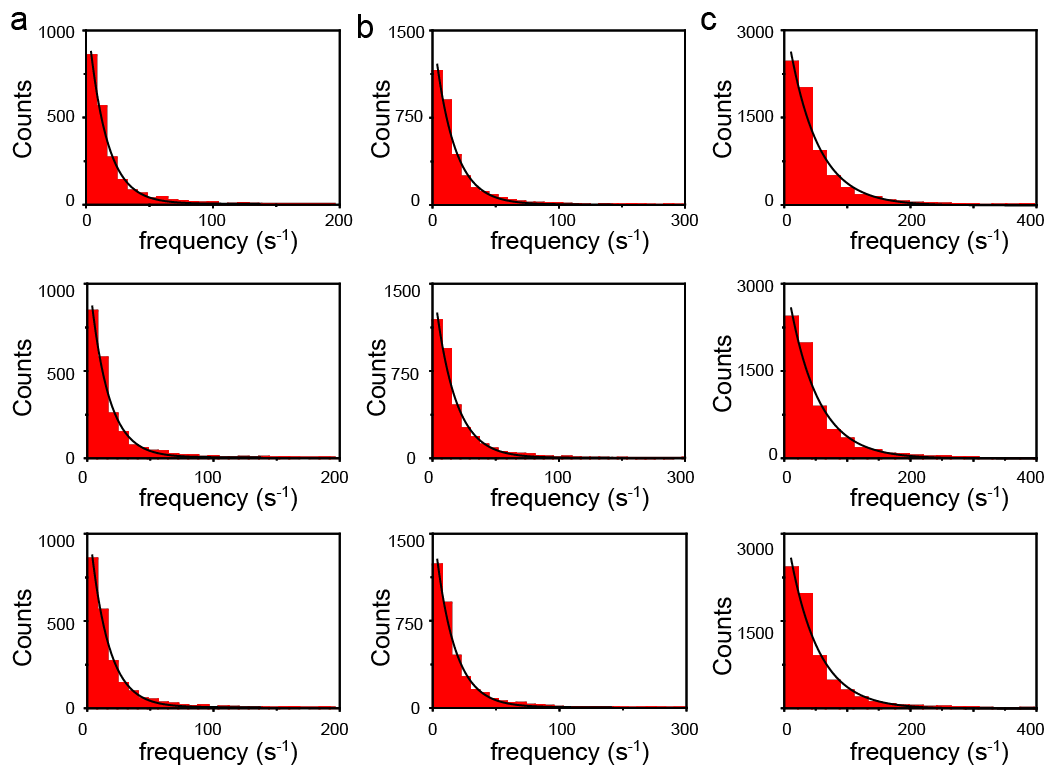


Figure S6. The frequency histogram of PNK-catalyzed reaction solution for the PNK concentration of: 0.01 U/uL (a), 0.05 U/uL (b), 0.1 U/uL (c) in the presence of 100 μM poly(dA_4_)-3’-P. The data was recorded in the 0-5 min (up), 5-10 min (middle) and 10-15 min (bottom), respectively. The event frequency was calculated by *f_r_* = 1/*t_on_*, where *t_on_* was the interval time between the consecutive typical blockage events. The histograms of frequency were fitted into the exponential equations.

Table S1. The event frequencies *f_r_* induced by the reaction buffer containing different PNK concentration.

| [PNK]  U/uL | Frequency (s-^1^)^*^ | | | | |
| --- | --- | --- | --- | --- | --- |
|  | 0-5 min | 5-10 min | 10-15 min | Average | SD |
| 0.01 | 14.44 | 14.50 | 14.54 | 14.47 | 0.04 |
| 0.05 | 23.33 | 24.01 | 22.81 | 23.38 | 0.49 |
| 0.10 | 46.18 | 46.34 | 46.03 | 46.18 | 0.13 |

^*^ The potential was set to +120 mV.


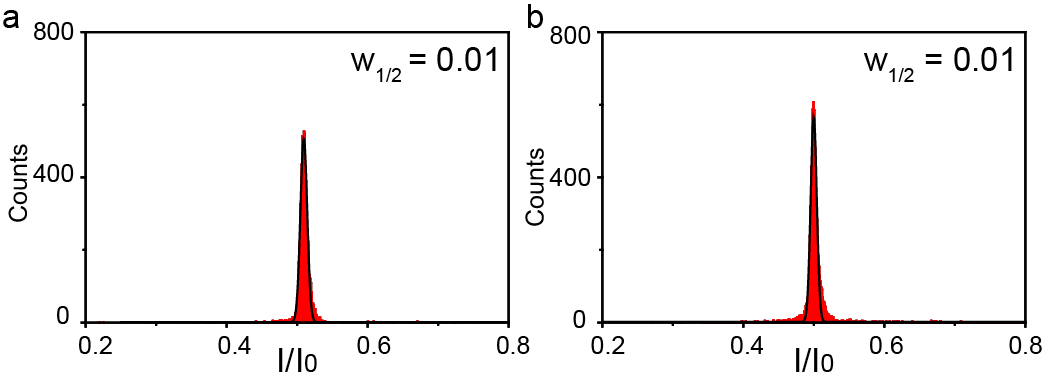


Figure S7. The histogram of I/I_0_ of poly(dA)_4_ (a) and the PNK-catalyzed events (b). The histograms of I/I_0_ were fitted into the Gaussian equations and their peak widths at half height were of I/I_0_ = 0.01.

**
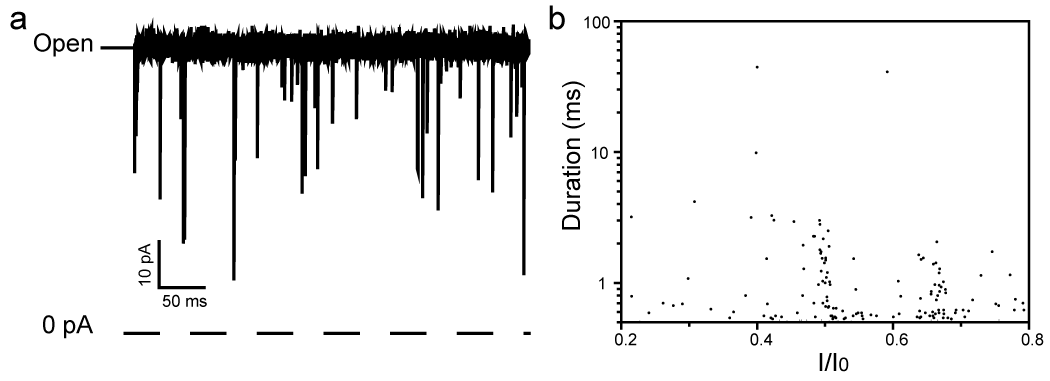
**

**Figure S8.** The raw data (a) and relative scatter plots (b) before adding the PNK in the real-time monitoring of PNK-catalyzed dephosphorylation assay. The recording time of data in scatter plots is 5 minutes.


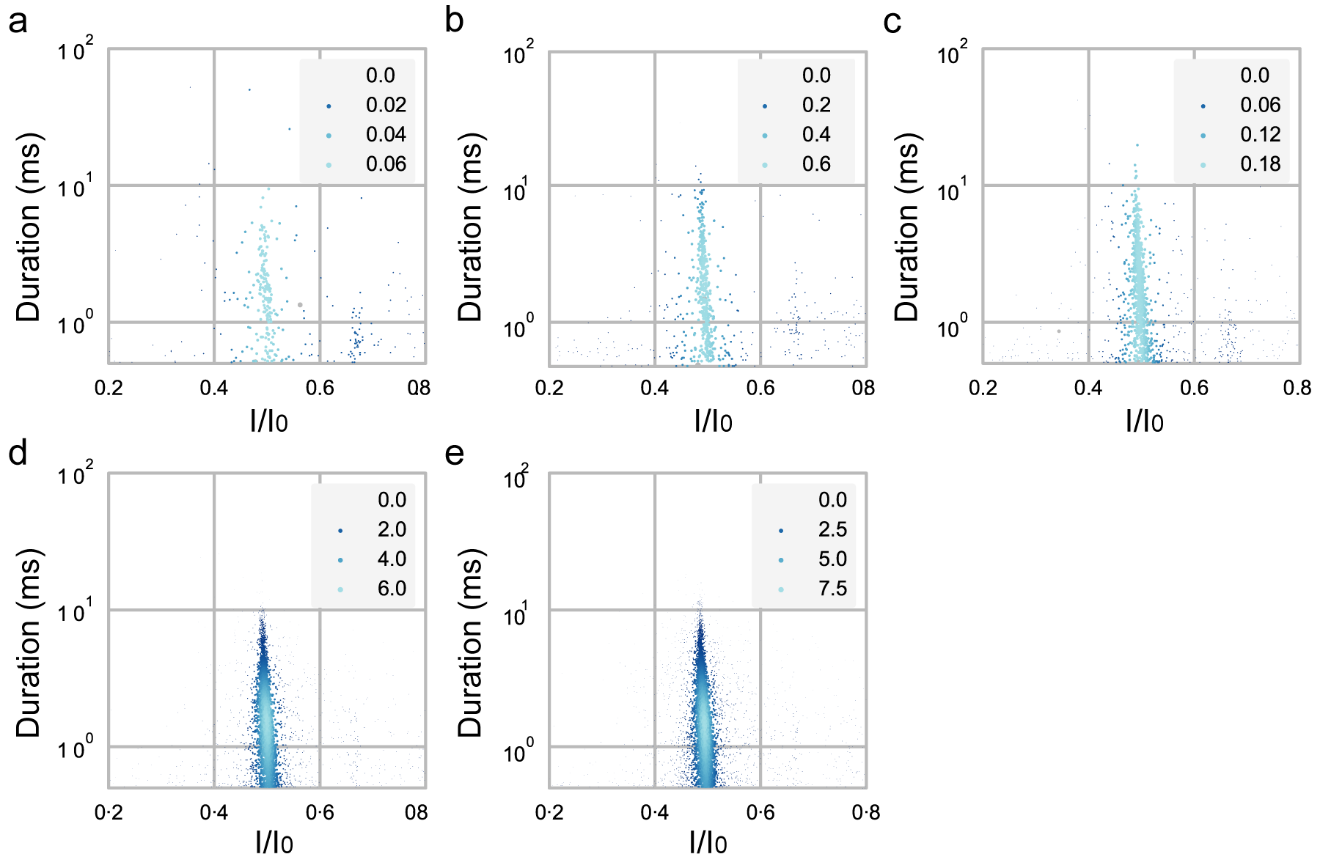


Figure S9. The scatter plots for real-time monitoring PNK dephosphorylation process by the aerolysin nanopore at the recording time of 0 h (a), 1 h (b), 2 h (b), 3 h (c), 4 h (d) and 5 h (e). Each scatter plots contain the statistical data of 8 minutes. Each point in the scatter plots is colored by the Kernel Density. 250 Units PNK, 100 μM poly(dA_4_)-3’-P were added into the cap side chamber which was filled with detection buffer (1.0 M KCl, 10 mM MgCl_2_ and 10 mM Tris, pH=6). The temperature was controlled at 30 ± 1 ℃ and the recorded potential was set at +120 mV.

**References**

1. Wang, Y. Q., Li, M. Y., Qiu, H., Cao, C., Wang, M. B., Wu, X. Y., Huang, J., Ying, Y.-L.; Long, Y.-T. (2018). Identification of Essential Sensitive Regions of the Aerolysin Nanopore for Single Oligonucleotide Analysis. Anal. Chem. *90*, 7790-7794.

2. Wu, X. Y., Wang, M. B., Wang, Y. Q., Li, M. Y., Ying, Y.-L., Huang, J., Long Y.-T. (2019), precise construction and tuning of an aerolysin single-biomolecule interface for single-molecule sensing CCS Chem. *1*, 304–312.

3. Cao, C., Ying, Y.-L., Hu, Z. L., Liao, D. F., Tian, H., & Long, Y.-T. (2016). Discrimination of oligonucleotides of different lengths with a wild-type aerolysin nanopore. Nat. Nanotechnol. *11*, 713-718.

4. Cao, C., Liao, D. F., Yu, J., Tian, H., Long, Y. T. (2017). Construction of an aerolysin nanopore in a lipid bilayer for single-oligonucleotide analysis. Nat. Protoc. *12*, 1901-1911.

5. Balijepalli, A., Ettedgui, J., Cornio, A. T., Robertson, J. W., Cheung, K. P., Kasianowicz, J. J., and Vaz, C. (2014). Quantifying short-lived events in multistate ionic current measurements. ACS Nano*8*, 1547-1553.
